# The Legal Past, Present and Future of Prenatal Genetic Testing: Professional Liability and Other Legal Challenges Affecting Patient Access to Services

**DOI:** 10.3390/jcm3041437

**Published:** 2014-12-15

**Authors:** Deborah Pergament, Katie Ilijic

**Affiliations:** Children’s Law Group, Case Western Reserve University School of Law, LLC, 142 East Ontario, Suite 525 Chicago, IL 60611, USA; E-Mail: dpergament@childrenslawgroup.com

**Keywords:** legal status of prenatal testing, ethics of prenatal testing, professional liability, genetic counseling, informed consent, cell-free fetal DNA testing, noninvasive fetal testing, preimplantation genetic diagnosis, termination of pregnancy, embryonic therapy, fetal therapy

## Abstract

This chapter is an overview of the current status of the law in the United States regarding prenatal genetic testing with an emphasis on issues related to professional liability and other challenges affecting patient access to prenatal genetic testing. The chapter discusses the roles that federal regulations, promulgated by the Centers for Medicare and Medicaid Services (CMS), the Food and Drug Administration (FDA) and the Federal Trade Commission (FTC), play in the regulation of prenatal genetic tests. The chapter discusses tort litigation based on allegations of malpractice in the provision of prenatal genetic testing and how courts have analyzed issues related to causation, damages and mitigation of damages. The chapter provides reference information regarding how individual states address causes of action under the tort theories of wrongful birth and wrongful life. The chapter concludes with a discussion of future legal issues that may affect clinical prenatal genetic testing services arising from the continued expansion of prenatal genetic testing, legal restrictions on access to abortion and the potential development of embryonic treatments.

## 1. Introduction

This chapter is a general overview of the current status of the law in the United States regarding prenatal genetic testing with an emphasis on issues related to professional liability and other challenges resulting from the current legal climate that affects patient access to prenatal genetic testing. It includes a discussion of future legal issues that may arise from the continued expansion of prenatal genetic testing and the potential development of embryonic treatments and expansion of fetal surgical and other interventions.

The chapter includes a discussion based on a definition of prenatal testing that incorporates carrier screening, preimplantation genetic diagnosis (PGD), invasive testing and noninvasive prenatal testing (NIPT). The chapter acknowledges the important discourse surrounding the complex intersection of laws, public policy, professional standards and social attitudes regarding reproductive rights and people with disabilities. However, the chapter does not take a position on any issue resulting from the long-standing debates over the legal and public policy implications arising from abortions after prenatal testing reveals fetal abnormalities or genetic disorders. As this chapter is intended only as an overview, it does not include all applicable statutory or case law, nor should it serve as legal advice. Any healthcare professional who has questions about a specific situation or the applicable laws should consult with an attorney on an individual basis.

In the United States, there is no single overarching legal authority dictating requirements or limitations on prenatal testing or the clinical practices that surround how healthcare professionals offer screening or explain or treat genetic disorders. The legal status of prenatal genetic clinical activities reflects the requirements enunciated by federal regulatory agencies, private health insurance practices and reimbursement policies, state professional licensure and health regulation laws. The practice and ethics guidelines promulgated by professional and scientific societies (e.g., the International Society for Prenatal Diagnosis, the National Society of Genetic Counselors, the American College of Obstetrics and Gynecology, the American College of Medical Genetics and Genomics) help structure clinical activities by providing guidelines and resources regarding best practices. These guidelines may influence the development of regulatory requirements and may be cited in medical malpractice lawsuits but, they are merely advisory and have no binding legal authority. The clinical practices surrounding prenatal genetic testing also may reflect both the anxieties that surround the possibility of civil litigation based on allegations of malpractice and the outcomes of such litigation.

## 2. Federal Regulations

Advances in genomic medicine frequently outpace the legal responses to the clinical provision of genetic tests, including the introduction of new prenatal testing technologies and the regulations imposed by government agencies on providers of clinical services. Three federal agencies play roles in the regulation of genetic tests: the Centers for Medicare and Medicaid Services (CMS); the Food and Drug Administration (FDA); and the Federal Trade Commission (FTC) [1]. The FTC focuses its regulatory authority on how tests are advertised and has the authority to regulate advertising that relates to health-related information directed at consumers to ensure that it is not false or misleading. With the advent of NIPT, direct-to-consumer marketing or marketing to healthcare providers without specialized training in genetics may give rise to greater involvement of the FTC in regulating the clinical prenatal testing. At this time, the CMS and FDA are the primary federal agencies addressing these issues, as reflected in the scope of the following discussion.

## 3. Clinical Laboratory Improvement Amendments of 1988 (CLIA)

The CMS has the authority to regulate clinical laboratories performing genetic tests to ensure compliance with the Clinical Laboratory Improvement Amendments of 1988 (CLIA) [2]. Congress passed CLIA in response to concern about the quality of clinical laboratory testing. This regulation expanded the Department of Health and Human Services’ existing authority to regulate clinical laboratories to include any clinical laboratory that examines “materials derived from the human body for the purpose of providing information for the diagnosis, prevention, or treatment of any disease or impairment of, or the assessment of the health of, human beings” [2]. The objective of CLIA is to certify the clinical testing quality, and verify procedures uses and the qualifications of the technical personnel processing the tests. In general, compliance with CLIA regulates the release of information. Laboratories are exempt from CLIA requirements if they are “research laboratories that test human specimens but do not report patient specific results for the diagnosis, prevention or treatment of any disease or impairment of, or the assessment of the health of individual patients” [2]. Whether a laboratory in the United States is required to comply with CLIA depends on the types of results the laboratory reports to participants. For example, laboratories that report individual-level results to research participants may have to comply with CLIA even if they do not charge for the testing or if the testing has occurred under the auspices of a research study.

The current CLIA requirements include performing ongoing measures to verify the accuracy of tests and procedures, usually through proficiency testing programs and establishing quality systems for testing processes and general laboratory systems. Laboratories performing moderate and high complexity tests also must have personnel and consultants with specific expertise available to fulfill responsibilities related to test procedures, interpretation and reporting of results, verification and other quality control measures. Laboratories that perform unmodified FDA-cleared or approved test systems must demonstrate that they can obtain performance speculations within the parameters established by the manufacturer. When using modified FDA cleared or approved test systems, the laboratory must establish performance specifications for accuracy, precision, analytical sensitivity, reporting ranges, reference intervals and other test performance criteria. Laboratories certified under CLIA must permit CMS or CMS agents to inspect the laboratory to assess its compliance with CLIA requirements [2].

Whether a laboratory must comply with CLIA depends on the nature and complexity of the tests being performed. As determined by the FDA, laboratory tests are classified as waived if they: are simple laboratory tests that pose no reasonable risk of harm if performed incorrectly; moderate and high complexity tests; and, specific specialty testing areas [2,3]. There is no specific category for genetic testing, but clinical cytogenetics falls into the specific specialty and subspecialty classification; and most molecular genetic tests are classified as moderate or high complexity. This requires laboratories to meet basic criteria for high complexity tests, but does not impose additional requirements. Such laboratories can meet these requirements through a Certificate of Compliance by CMS or a Certificate of Accreditation from an approved accreditation organization (e.g., the College of American Pathology). In reality, there are no specified proficiency testing requirements for genetic testing laboratories, because genetics is not a designated specialty area and none of the specified regulated analytes include nucleic acids (*i.e.*, RNA and DNA). The Centers for Disease Control and Prevention’s Clinical Laboratory Improvement Advisory Committee recommended adding a genetic specialty under CLIA, and CMS considered, but eventually decided against such an action [4]. The decision was justified, in part, because of the potential lack of sufficient proficiency samples for many genetic tests and the lack of adequate data to assess clinical validity.

CLIA has several means of enforcement, including cancellation of a laboratory’s approval to Medicare payments and limiting Medicaid payments. In addition, civil monetary penalties may be imposed upon laboratories in amounts up to $10,000 per day of non-compliance or per violation. If the CMS has reason to believe that continuation of a laboratory’s activities would constitute a significant hazard to the public health, it may bring suit in a federal court of competent jurisdiction [2].

States also may have separate licensing requirements, and these licensing requirements may provide exemption from CLIA requirements if they are as or more stringent than the ones imposed by CLIA. Only New York and Washington State have satisfied these criteria. The New York Clinical Laboratory Evaluation Program requirements apply to all laboratories that conduct clinical testing on specimens originating in New York State, including out-of-state facilities that accept such specimens from New York healthcare providers [5]. The New York requirements exceed the CLIA standards with the implementation of specific genetic testing standards, which laboratories seeking a genetic testing permit must satisfy. These requirements may serve as a model for amplification and refinement of the federal CLIA with regards to genetic testing. Laboratories that receive New York State permits must demonstrate that they obtain the subject’s informed consent or the consent of a legally authorized decision maker. Reports must also include a statement of and interpretation of its findings, specify the test’s technical limitations, and provide information about possible additional testing and recommendations for referral to a genetic counselor depending on the situation and the test methodology, including a list of all variants examined by the assay [5].

## 4. Food and Drug Administration: Genetic Test Kits

In theory, the FDA has broad authority to regulate the safety and effectiveness of genetic tests as medical devices under the Federal Food, Drug, and Cosmetic Act (FFDCA). The FDA can regulate genetic tests sold to laboratories, but has not exercised this authority over all testing kits and related materials that are used in carrier screening or prenatal diagnosis [6]. The oversight the FDA can exercise over a genetic test depends on the intended use and the risks posed by an inaccurate test result.

The FDA categorizes medical devices, including genetic tests, into three categories ranging from Class I (relatively low risk products) to Class III (products subject to the greatest level of scrutiny) [6]. Whether the FDA regulates a test is determined by how it enters the market place: a test marked as a commercial test “kit”, such as a group of reagents used in the processing of genetic samples that are packaged together and sold to multiple laboratory facilities [6]; a test that comes to market as a laboratory-developed test (LDT) under the auspices and use of a single laboratory is subject to “enforcement discretion”. The FDA has historically regulated tests sold as kits and practiced enforcement discretion for LDTs.

In June, 2010, the FDA announced that it decided to exercise its authority over all LDTs [7]. The decision was justified by a number of reasons, including that the public needs assurance that LDTs are sound and reliable. Clinical laboratories and manufacturers of LDTs are actively opposed to regulation of LDTs by the FDA. In June, 2013, the American Clinical Laboratory Association (ACLA) filed a citizen petition under the FFDCA requesting that the FDC refrain from issuing draft or final guidance or rules purporting to regulate LDTs as devices [8]. The ACLA has argued that the FFDCA does not provide for the statutory authority to regulate LDTs, as they are proprietary procedures not subject to regulation under the FFDCA [8]. The ACLA also asserts that LDTs do not meet the definition of commercial distribution, which requires that a product be delivered, distributed or placed on the market. The FDA has not yet finalized guidance with respect to all LDTs [8].

Prenatal testing, including new methods using cell-free fetal DNA-based NIPT, is not currently regulated by government agencies, and there is only limited regulation of the test kits and materials used in these processes. Professional guidance is still being developed and stresses that in the absence of regulation, companies and clinicians should endeavor to develop responsible and ethical best practice guidelines. The absence of specific regulations regarding test kits and other laboratory material may not continue. Intellectual property disputes affecting the patenting and licensing landscapes of these technologies, and the current central role commercialization plays in the implementation of these technologies, may result in efforts to impose regulations on testing materials [9]. Moreover, the current political climate (further discussed below) resulting in the intersection of prenatal genetic testing and abortion politics may constrict widespread access by imposing regulations on the advertising regarding tests and laboratory materials used to provide them.

## 5. Medical Malpractice

It can be argued that one of the most significant influences on the actions of physicians and allied health professionals providing prenatal genetic testing services is tort litigation or fear of tort litigation. When a child is born with a genetic disorder after prenatal genetic testing, the parents may seek financial recourse through the legal system for costs associated with custodial care, past and future medical expenses and compensation for alleged emotional damages.

Healthcare professionals facing litigation express unease about their continued ability to provide care for other patients and the taint on their professional reputations as clinicians and researchers. In addition, some professionals providing services in privately maintained facilities are concerned about the financial impact, especially if the jurisdiction allows for recovery of damages in excess of malpractice insurance policy limits. In the past two years, three cases have given rise to multi-million dollar verdicts; *Levy v. Legacy Health System* (2012) resulted in a $2.9 million dollar verdict by an Oregon jury and a later confidential settlement for an undisclosed amount. This case involved a patient who underwent CVS and allegations of failure to diagnosis mosaic Down syndrome [10]. Another case heard in Tennessee, *Paule v. Hughes and Genesis Genetics* (2012), resulted in a jury verdict for over $13 million. The plaintiffs alleged that improperly performed PGD for cystic fibrosis in a twin pregnancy resulted in the birth of one affected and one unaffected infant [11]. Another recent case, *Wuth v. Lab Corp.* (2013), resulted in a $50 million dollar verdict in Washington State for failure to provide genetic counseling and diagnosis an unbalanced translocation [12]. These large verdicts are dramatic, but not typical of cases filed alleging negligent prenatal genetic testing. Only a handful of suits seeking damages based on claims of medical malpractice in the provision of prenatal testing are filed each year in the United States and even fewer result in a trial [13]. Many such cases are dismissed on preliminary motions or are settled out of court with costs being paid through malpractice insurance [13]. Settlements, usually under court seal or pursuant to a confidentiality agreement, may occur even in cases that originally resulted in large jury verdicts, particularly if factual or legal issues arose during the trial that could support overturning the judgment on appeal.

Despite the limited numbers of actual cases, concerns about liability regularly guide clinical activities intended to facilitate informed decision-making by patients, such as the provision of literature explaining the risk of error and associated consent forms. The focus is on avoiding allegations that the healthcare provider was negligent because of omission or failed communication of pertinent information or failure to provide information about the potential for error of diagnostic information. Concerns about potential lawsuits may also affect how healthcare providers counsel their patients regarding the options available for affected pregnancies. These concerns have been intensified by the clinical challenges created by rapidly-evolving prenatal genetic testing technologies and laws surrounding the provision of reproductive healthcare. In recent years, the introduction of preimplantation diagnostics, array-based cytogenetic assays and single-cell sequencing, NIPT utilizing cell-free DNA (cfDNA) and the potential of research on the isolation of nucleated fetal cells from maternal blood have arguably raised expectations that prenatal testing should be risk and error free.

As in most medical malpractice cases, most cases involving prenatal testing are brought under theories of negligence. The plaintiffs typically allege: that the healthcare professional failed to provide services within the standard of reasonable professional practice in the healthcare provider’s profession or specialty that existed at the time the care in question was rendered; that the healthcare professional failed to act in accordance with such a standard; and, as a proximate result, the injured person suffered injuries that would not otherwise have occurred. The majority of states define professional standard of care with regard to medical malpractice cases as the standard of professional conduct that would be expected from a reasonable physician [13]. A minority of states consider the standard of care as measured by the reasonable patient under which the physician is expected to disclose any risks and alternatives that may be material to enable an informed decision by a reasonable person [13].

Cases alleging malpractice in the provision of prenatal genetic testing arise in a number of contexts. These typically include allegations that a physician or genetic counselor was negligent in the provision of genetic counseling by failing to provide accurate information about potential reproductive risk based on age or carrier status, refusing requests to perform invasive procedures or failing to inform the patient about the need or availability of such procedures. Allegations that the physician was negligent in the selection of the laboratory that analyzed the genetic material or failed to interpret the test results correctly are also common. Other cases have been brought against laboratories alleging that carrier testing or invasive testing was negligently performed or the results were misinterpreted. Another fact pattern involving allegations of laboratory negligence or genetic counseling based on misinterpreted laboratory results involves plaintiffs claiming they would not have conceived if a previously born child had been correctly diagnosed with the condition that affected subsequent pregnancies or if they had been properly counseled about the risks of conceiving another affected child. These are commonly referred to as “wrongful birth” or “wrongful life” cases.

The controversy surrounding wrongful birth and wrongful life litigation has existed for many years and has been well documented. A large body of legal literature has been developed. The literature encompasses debates over the legal elements that must be demonstrated, the strategies involved in proving or defending such causes of actions and a myriad of ethical and psychosocial questions that arise from both individual cases and tort liability, in general, for failure to diagnose prenatally genetic conditions. These include issues related to the right to abortion, the interplay between prenatal diagnosis and the social status of people with disabilities, and the roles tort liability and awards for damages should play in addressing the needs of people with disabilities [14]. Courts faced with these cases have overwhelmingly rejected wrongful life actions, while allowing wrongful birth actions. Legislative enactments regarding whether such allegations are permissible under states’ rules of procedure also reflect a similar pattern. 

The earliest cases asserting theories of wrongful birth and wrongful life did not involve genetic testing, but arose from the births of healthy children who were, for a variety of reasons, unexpected or unwanted by their parents and were termed “wrongful pregnancy” or “wrongful conception”. Wrongful conception is an action based on the assertion that the birth of a healthy, but unwanted, child results from the defendant’s negligence. The majority of cases involving prenatal testing are brought by parents either as individuals or on behalf of their children as their child’s next friend (one acting for the benefit of a minor child or other person unable to look after his or her own interests or manage his or her own lawsuit). Parents alleging that they have been injured by the birth of a child with a genetic disorder typically do so under theories of negligence, lack of informed consent, wrongful pregnancy/wrongful conception, wrongful birth or wrongful life, depending on the facts of the case and the jurisdictions controlling statutory and case law. Generally, wrongful life is a claim brought on behalf of a child for damages resulting from being born with a defect due to the defendant’s negligence.

## 6. Wrongful Life

Wrongful life claims are typically brought by a parent or a guardian on behalf of a minor child with a genetic or congenital disorder seeking to recover damages for the healthcare provider’s alleged negligence, either in the pre-conception or prenatal period, to predict or diagnose the child’s genetic disorder [15]. The complaints in these cases usually contain allegations that the defendant healthcare professional failed to accurately perform carrier screening or other genetic tests, to inform the child’s parents about the genetic risks associated with certain disorders, to accurately advise the child’s parents about the risks associated with pregnancy of an affected fetus and subsequent childbirth, or to perform a surgical procedure intended to prevent the birth of a child with a genetic disorder. 

The premise of prenatal diagnosis is that knowing the health status of the fetus will enable expectant women and their partners to make rational reproductive decisions. The principles of non-directive counseling emphasize unbiased information and assistance. In comparison, cases alleging malpractice in the provision of prenatal genetic testing force courts to examine issues related to the legal implications of finding that the alleged negligence denied the plaintiffs the opportunity to avoid the conception or birth of a child with a disability. A wrongful life case poses the fundamental question of whether the child would have been better off not having been born. The parents’ claims are based on the general assertion that, as a result of negligence, they were deprived of the right to make rational, autonomous decisions about their offspring. The overarching question in many cases, regardless of which theory is being asserted, is whether the healthcare professional failed to disclose information that was material or could have influenced the patient’s decision making process. 

Accordingly, the legal theories under which these cases are brought have forced the courts to examine a myriad of issues not only related to abortion, but the appropriateness of recognizing the right to seek damages for a life affected by disability when the alternative is the termination of an affected pregnancy. As one court commented in a case involving a wrongful birth claim against physicians who had provided genetic counseling and misinformed the mother that she had a “very low” risk of being a carrier for hemophilia:

“In a wrongful life case, the child does not assert the negligence of the defendants caused his inherited or congenital abnormality, that the defendants could have done anything that would have decreased the possibility that he would be born with such defects, or that he ever had a chance to be normal. The essence of the child’s claim is that the medical professional’s breach of the applicable standard of care precluded an informed parental decision to avoid his conception or birth. But for this negligence, the child allegedly would not have been born to experience the pain and suffering attributable to his affliction”.

[16]

The controversy surrounding the ethical and moral concerns that arise from these cases is apparent in the widespread rejection of this cause of action by courts. Some courts side-step this issue by holding that the wrongful pregnancy, wrongful birth and wrongful life actions are not “new torts”, but fall within the traditional boundaries of negligence actions. These courts, in sustaining the plaintiffs right to pursue litigation, focus on the defendant’s alleged negligence and not on the issues related to arguments over the sanctity of human life or the propriety of addressing issues related to assessing damages based on valuations of a child’s life, with or without disability. As the courts in *Reed v. Campagnolo* (1993), *Viccaro v. Milunsky* (1990) and *Liddington v. Burns* (1995) noted, any “wrongfulness” lies not in the life, the birth, the conception or the pregnancy, but the negligence of the physician [17,18,19]. “The harm, if any, is not the birth itself but the effect of the defendant’s negligence on the parents resulting from the denial of their right to decide whether to bear a child or whether to bear a child with a genetic or other defect [18]”.

While other courts confront the ethical issues surrounding questions about the nature and value of human life, with or without disability, directly, courts have overwhelmingly rejected wrongful life actions, finding that they are not able to evaluate claims requiring the calculation of damages based on comparisons of the value of life with a disability and the value of non-existence. One of the most frequently cited cases, *Becker v. Schwartz* (1978), involved allegations that the mother’s physician failed to advise her of the increased risk of birth defects in pregnancy of women over thirty-five years of age and failed to recommend amniocentesis [20]. The child was affected with Down syndrome and the parents sued, alleging wrongful birth and wrongful life. The court found that the action for wrongful life was fundamentally flawed, because it could not be demonstrated that the child suffered legally cognizable injury, because there is no right to “be born as a whole, functional human being” [20]. In casting this issue as a philosophical construct, the court described calculating damages as “dependent upon a comparison between the Hobson’s choice of life in an impaired state and nonexistence. This comparison the law is not equipped to make” [20]. A frequently quoted part of the decision formulates this argument:

“Whether it is better never to have been born at all than to have been born with even gross deficiencies is a mystery more properly to be left to the philosophers and the theologians. Surely the law can assert no competence to resolve the issue, particularly in view of the very nearly uniform high value which the law and mankind has placed on human life, rather than its absence”.

[20]

In keeping with this analysis, state legislative enactments addressing the right of action under wrongful life typically have prohibited this cause of action. In fact, only three states, Washington, New Jersey and California, currently recognize these claims [21,22,23]. The majority of states with specific statutes addressing the issue explicitly prohibit wrongful life claims and several also prohibit wrongful birth claims. Table 1 provides applicable legal citations regarding how courts and/or legislatures in each of the 50 United States have considered wrongful birth and wrongful life causes of action.

## 7. Wrongful Pregnancy/Wrongful Birth

The earliest cases involving parents making claims of wrongful birth or wrongful pregnancy were based on allegations that the parents had been harmed by the birth of a healthy child, who, for a variety of reasons, was unexpected or unwanted by the parents [24,25,26]. In cases alleging wrongful pregnancy (also referred to as wrongful conception), the plaintiffs typically asserted they undertook active measures to avoid pregnancy by means such as undergoing surgical sterilization or abortions or had used faulty contraceptive products. In such cases, the case of action arose as a result of the conception and birth of a normal, healthy child. The parents then sought to recover damages for the pain and suffering associated with the ineffective sterilization procedure, the unwanted pregnancy and birth and the loss of service and consortium. The parents may have also attempted to recover damages for medical expenses and the ordinary expense of raising a normally developing child to adulthood. The outcome of such cases, resulted from factors that often influence the outcome of litigation regardless of the overarching legal theory of the case. Success for the plaintiffs is dependent on both the common and statutory law that governs in the jurisdiction, the facts surrounding the case and state and local attitudes. In several cases, recovery was limited to the damages arising from the negligent sterilization procedure and prenatal and postnatal care, and in a rare instance, the parents recovered the ordinary costs of rearing a child without disabilities to adulthood [27,28,29,30]. There are also some cases alleging that a negligent sterilization procedure resulted in the birth of a child with a genetic disorder, and the parents may seek damages associated with the additional expenses of raising a child with a disability [31].

An important variation on this theory involving prenatal testing arises when parents sue for wrongful birth after seeking to avoid any pregnancy, but eventually having a child with disabilities, necessitating expenditures beyond the costs associated with rearing a child to adulthood. The overarching claim is that a physician or other healthcare provider did not cause the damage or disease in the fetus, but their negligence caused a loss of opportunity to prevent conception or live birth of an infant with a genetic abnormality. Such plaintiffs may claim they decided that they did not wish to have children because they were at risk for having a child affected by a genetic disorder or had already had a child with the disorder. In one such case, *Molloy v. Meier* (2004), the plaintiffs were able to overcome Minnesota’s statutory prohibition on wrongful birth and wrongful life claims because they alleged they would have foregone conception, not abortion. This case involved allegations of failing to interpret and report accurately laboratory results related to the diagnosis of Fragile X in an earlier born sibling and the subsequent conception and birth of another affected child [32].

Other cases may arise when parents were under the impression that the pregnancy was normal because their healthcare provider failed to inform them that prenatal tests were available or failed to provide information about known risks [33,34,35]. In such a case, the parents of a child born with a genetic disorder allege wrongful birth based on the assertion that the healthcare provider negligently failed to inform the parents, in a timely fashion, of the increased possibility that the mother would give birth to a child with a genetic disorder, thereby precluding an informed decision as to whether to have the child. This standard does not require a healthcare provider to identify every chance of the occurrence of every possible birth defect. The proof of causation is met if the plaintiff can show that, but for the defendant’s negligent failure to inform the mother of the risks of delivering a child with a genetic disorder, the mother would have obtained an abortion. As one court described, the parent claim of injury is for the lost choice over whether or not to carry a child with a disability to term as they “allege they would have avoided conception or terminated the pregnancy by abortion but for the negligence of those charged with prenatal testing, genetic prognosticating, or counseling parents to the likelihood of giving birth to a physically or mentally impaired child” [16].

## 8. Mitigation of Damages

The doctrine of “mitigation of damages” is also sometimes called the doctrine of avoidable consequences. In general, it requires the party injured by breach of tort duty to minimize damages. Mitigation of damage is an affirmative defense and applies when the plaintiff fails to take reasonable actions that would tend to mitigate the alleged injuries. In several cases arising out of claims of negligent provision of prenatal genetic testing, defendants have asserted that the parents had a duty to mitigate their damages by terminating the pregnancy. Another variation of this defense may arise in the context of cases involving PGD with the argument that parents have a duty to undergo confirmatory invasive testing, and if they do not, they have failed to mitigate potential damages.

In *Liddington v. Burns* (1995), an Oklahoma case, one defendant argued two inconsistent theories in an attempt to have the case dismissed upon motion for summary judgment [19]. The first was that he was precluded from liability for failing to provide an ultrasound because state statute immunized a defendant from civil liability for failure to perform medical procedures that result in an abortion. The court rejected the argument finding that ultrasounds are not medical procedures that result in an abortion [19]. In the alternative, the defendant argued that any negligence on his part was superseded by the mother’ failure to terminate in the third trimester after another physician in Arizona diagnosed the fetal anomalies. The court rejected this argument, too, finding that there was undisputed evidence that the mother could not have legally had a third-trimester abortion in Arizona and as a matter of law the mother’s failure to obtain a third trimester abortion did not relieve the defendant of liability for medical negligence, if any [19]. 

**Table 1:** Check-marks indicate that the cause of action is allowed in that state; “X” indicates that the cause of action is not allowed in that state.

State	Wrongful Birth Cause of Action	Wrongful Life Cause of Action
**Alabama**	√ (*L.K.D.H. v. Planned Parenthood of Alabama*, 944 So.2d 153 (Ala. Civ. App. 2006))	X (*Elliott v. Brown*, 361 So.2d 546 (Ala. 1978); *L.K.D.H. v. Planned Parenthood of Alabama*, 944 So.2d 153 (Ala. Civ. App. 2006))
**Alaska**	√ (*M.A. v. U.S.*, 951 P.2d 851 (Alaska 1998))	
**Arizona**	X (A.R.S. § 12-719) 1 (2012)	X (A.R.S. § 12-719)
**Arkansas**	X (*Brown v. Wyatt*, 202 S.W.3d 555 (Ark. Ct. App. 2005))	
**California**	√ (*Turpin v. Sortini*, 643 P.2d 954 (Cal. 1982))	√ (*Turpin v. Sortini*, 643 P.2d 954 (Cal. 1982))
**Colorado**	√ (*Lininger v. Eisenbaum*, 764 P.2d 1202 (Colo. 1988))	X (*Lininger v. Eisenbaum*, 764 P.2d 1202 (Colo. 1988))
**Connecticut**	√ (*Ochs v. Borrelli*, 445 A.2d 883 (Conn. 1982))	X (*Rich v. Foye*, 976 A.2d 819 (Conn. Super. Ct. 2007))
**Delaware**	√ (*Garrison v. Medical Center of Delaware Inc.*, 581 A.2d 288 (Del. 1989))	X (*Garrison v. Medical Center of Delaware Inc.*, 581 A.2d 288 (Del. 1989))
**Florida**	√ (*Kush v. Lloyd*, 616 So.2d 415 (Fla. 1992))	X (*Kush v. Lloyd*, 616 So.2d 415 (Fla. 1992))
**Georgia**	X (*Etkind v. Suarez*, 519 S.E.2d 210 (Ga. 1999))
**Hawaii**		
**Idaho**	X (I.C. § 5-334) 1 (2010)	X (I.C. § 5-334)
**Illinois**	√ (*Clark v. Children’s Memorial Hosp.*, 955 N.E.2d 1065 (Ill. 2011))	X (*Clark v. Children’s Memorial Hosp.*, 955 N.E. 2d 1065 (Ill. 2011))
**Indiana**	√ (*Chaffee v. Seslar*, 786 N.E.2d 705 (Ind. 2003))	X (*Cowe v. Forum Group, Inc.*, 575 N.E.2d 630 (Ind. 1991))
**Iowa**	√ (*Nanke v. Napier*, 346 N.W.2d 520 (Iowa 1984))	
**Kansas**	X (K.S.A. 60-1906) ^1^ (2013)	X (K.S.A. 60-1906)
**Kentucky**	X (*Grubbs v. Barbourville Family Health Center, P.S.C.*, 120 S.W.3d 682 (Ken. 2003))	X (*Grubbs v. Barbourville Family Health Center, P.S.C.*, 120 S.W.3d 682 (Ken. 2003))
**Louisiana**	√ (*Pitre v. Opelousas General Hosp.*, 530 So.2d 1151 (La. 1988))	
**Maine**	X (24 M.R.S.A. § 2931 (1990) for the birth of a healthy child) ^1^	X (24 M.R.S.A. § 2931 for the birth of a healthy child)
**Maryland**	√ (*Reed v. Campagnalo*, 630 A.2d 1145 (Md. 1993))	X (*Kassama v. Magat*, 792 A.2d 1102 (Md. 2002))
**Massachusetts**	√ (*Viccaro v. Milunsky*, 551 N.E.2d 8 (Mass. 1990))	X (*Viccaro v. Milunsky*, 551 N.E.2d 8 (Mass. 1990))
**Michigan**	X (M.C.L.A. 600.2971) 1 (2011)	X (M.C.L.A. 600.2971)
**Minnesota**	X (M.S.A. § 145.424) ^1^ (2005)	X (M.S.A. § 145.424)
**Mississippi**		
**Missouri**	X (V.A.M.S. 188.130) ^1^ (1986)	X (V.A.M.S. 188.130)
**Montana**		
**Nebraska**		
**Nevada**	√ (Greco v. U.S., 893 P.2d 345 (Nev. 1995))	X (Greco v. U.S., 893 P.2d 345 (Nev. 1995))
**New Hampshire**	√ (Smith v. Cote, 513 A.2d 341 (NH 1986))	X (Smith v. Cote, 513 A.2d 341 (NH 1986))
**New Jersey**	√ (Hummel v. Reiss, 608 A.2d 1341 (NJ 1992))	√ (Procanik v. Cillo, 478 A.2d 755 (NJ 1984))
**New Mexico**		
**New York**	√ (*Bani-Esraili v. Lerman*, 505 N.E.2d 947 (NY 1987))	X (*Becker v. Schwartz*, 386 N.E.2d 807 (NY 1978))
**North Carolina**	X (*Azzolino v. Dingfelder*, 337 S.E.2d 528 (NC 1985))	X (*Azzolino v. Dingfelder*, 337 S.E.2d 528 (NC 1985))
**North Dakota**		X (NDCC 32-03-43) (1985)
**Ohio**		X (*Hester v. Dwivedi*, 733 N.E.2d 1161 (Ohio 2000))
**Oklahoma**	X (63 Okl.St.Ann. § 1-741.12) ^1^ (2010)	X (63 Okl.St.Ann. § 1-741.12)
**Oregon**		
**Pennsylvania**	Statute prohibiting cause of action was found unconstitutional (See *Sernovitz v. Dershaw*, 57 A.3d 1254 (Pa. 2012))	Statute prohibiting cause of action was found unconstitutional (See *Sernovitz v. Dershaw*, 57 A.3d 1254 (Pa. 2012))
**Rhode Island**	√ (*Emerson v. Magendantz*, 689 A.2d 409 (RI 1997))	
**South Carolina**		X (*Willis v. Wu*, 607 S.E.2d 63 (SC 2004))
**South Dakota**	X (SDCL 21-55-2) ^1^ (1987)	X (SDCL 21-55-2)
**Tennessee**	(*Smith v. Gore*, 728 S.W.2d 738 (Tenn. 1987))	
**Texas**	√ (*Nelson v. Krusen*, 678 S.W.2d 918 (Tex. 1984))	X (*Nelson v. Krusen*, 678 S.W.2d 918 (Tex. 1984))
**Utah**	X (*Wood v. University of Utah Medical Center*, 67 P.3d 436 (Utah 2002))	Statute prohibiting was repealed by Laws 2008, c. 3, § 1474, effective February 7, 2008
**Vermont**		
**Virginia**	√ (*Naccash v. Burger*, 290 S.E.2d 825 (Va. 1982))	
**Washington**	√ (*Harbeson v. Parke-Davis, Inc.*, 656 P.2d 483 (Wash. 1983))	√ (*Stewart-Graves v. Vaughn*, 170 P.3d 1151 (Wash. 2007))
**West Virginia**	√ (*James G. v. Caserta*, 332 S.E.2d 872 (W.V. 1985))	X (*James G. v. Caserta*, 332 S.E.2d 872 (W.V. 1985))
**Wisconsin**	X (*Slawek v. Stroh*, 215 N.W.2d 9 (Wis. 1974))	X (*Slawek v. Stroh*, 215 N.W.2d 9 (Wis. 1974))
**Wyoming**	√ (*Beardsley v. Wierdsma*, 650 P.2d 288 (Wyo. 1982))	X (*Beardsley v. Wierdsma*, 650 P.2d 288 (Wyo. 1982))

^1^ Statutes prohibiting “wrongful birth” claims have the practical effect of legalizing a doctor’s ability to withhold information from patients if the doctor feels that withholding such information could prevent an abortion. A patient cannot sue the doctor for malpractice based on the fact that but for a wrongful omission, the birth would have been prevented.

Courts have considered whether the cause of action should stand in light of evidence that the parents did not terminate the pregnancy. For example, the court in *Davis v. Board of Supervisors of Louisiana State University* (1998) noted that the parents had decided not to terminate the pregnancy at 22–23 weeks gestation, because the mother believed the fetus was viable [36]. The defendants offered evidence that the fetus was not viable until 26 or 27 weeks. The court dismissed the claim, in part, because the defendants sustained their burden that their actions were not the cause in fact of the parents’ decision not to abort and that uncontroverted evidence demonstrated that the option of an abortion in Louisiana was just as available to the parents at 23 weeks as it was at 16 weeks [36]. Similarly, in a New Hampshire case involving allegations of diagnosis partial trisomy 9q, the court ruled that parents do not have a viable claim if the mother could have had an abortion when the parents were told, after an amniocentesis, that the karyotype was normal, but later informed at 23–24 weeks that the fetus was possibly severely affected [37]. In finding that the defendants made a timely disclosure of the possibility of birth defects and dismissing the case, the court stated

“We also acknowledge that a wrongful birth claim is unlike any other medical malpractice action because it involves the uniquely personal choice to terminate a pregnancy or give birth to a child with the increased possibility of severe birth defects. In this respect, a fact finder should also consider the plaintiff’s emotional and physical ability to digest and act upon the information concerning the increased possibility of birth defects within the time period at issue, as well as her willingness and ability to travel to another jurisdiction to obtain an abortion during her third trimester, had she been able to arrange one”.

[37]

The most recent judicial considerations focus on issues related to calculation of damages that parents may incur resulting from the birth of a child with a genetic disorder and the expenses associated with the care of that child.

## 9. Damages

The success of efforts to recover for emotional damages based on allegations of negligence in genetic counseling or interpretation of prenatal testing depends on the court’s consideration of the legal theory underlying the claims of emotional distress. There have been decisions that rejected the parents’ claims of emotional distress based on the reasoning that the parents suffered no physical or mental anguish other than the anguish of their child as a result of the presumed negligence by the defendant, nor did the negligence directly cause the child to fall victim to the disease [38]. Some courts have allowed recovery based on a theory that emotional distress results from being denied the opportunity to decide whether to conceive a child [39,40]. In these cases, the court views the allegations under the theory that the healthcare provider’s negligence was the proximate cause of the plaintiff’s emotional pain and suffering. A determination of whether an act is the proximate cause of an injury encompasses both “cause in fact” and “legal cause”. An injury or damage is considered proximately caused by negligence when it appears from the evidence that the act or omission played a substantial role in causing the actual injury or damage and that the injury or damage was either the direct result or was a reasonably probable consequence of the act or omission [41].

Some courts have refused to allow parents to recover damages resulting from negligent infliction of emotional distress, reasoning that the “zone-of-danger” rule prevents the finding of liability [42]. The “zone-of-danger” rule limits the liability of persons accused of negligent infliction of emotional distressed based on the theory that recovery for damages can only occur if the plaintiff were placed in immediate risk of physical harm by the defendant’s negligence and frightened by the risk of harm [43]. In disallowing recovery based on claims of negligent infliction of emotional distress in wrongful birth cases, courts have held that parents do not fall within the “zone of danger”, because the healthcare provider’s negligence did not physically endanger the parents of the child born with the genetic disorder [42].

There has been, however, a recent reconsideration of this theory within the context of wrongful birth cases. In 1986, the Illinois Supreme Court sustained a cause of action for “wrongful birth” in case involving allegations of negligent genetic counseling related to inaccurate risk assessment for hemophilia [16]. The court found that the parents could seek damages for extraordinary expenses, such as medical, hospital, institutional, educational and costs otherwise necessary to properly manage and treat the congenital or genetic disorder, but dismissed the parents’ claims for negligent infliction of emotional distress, because they had failed to plead facts demonstrating that they fell within the “zone-of-danger” [16]. A recent case involving failure to diagnose Angelman syndrome in the plaintiffs’ first child and the subsequent conception of an affected sibling illustrates the emerging jurisprudence. In that case, the Illinois Supreme Court held that the “zone-of-danger” no longer applies to wrongful birth cases or other cases in which a tort has already been committed against the plaintiffs and they allege emotional distress as an element of damages for that tort [42]. This interpretation makes sustaining a cause of action for negligent infliction of emotional distress in cases involving wrongful birth more likely. Yet, it is important to consider that the finding of liability is highly fact specific and dependent on many variables, including whether the plaintiff can credibly demonstrate that, but for the defendant’s negligence, she would have terminated an affected pregnancy.

Another area of significant controversy regarding potential damages in wrongful birth cases concerns the interplay between the benefits and burden of having a child, the parental responsibility for the care of children and adult offspring with disabilities and whether available public resources negate any claims for damages. Some courts have examined wrongful birth cases under an analysis that focused on the burden and benefits of raising an unwanted child or a child with disabilities on the parents [43]. Under the “benefits rule”, the court weighs the burdens imposed on the parents against the benefits conferred by the child [43].

In one case, involving failure to diagnose Down syndrome, the court found that the defendants were entitled to summary judgment of the case, because they established that the child was generally in good physical health and that his educational and developmental needs will be provided for at no cost, under government programs, such as those available pursuant to the Individuals with Disabilities Education Act [44]. The court also found that the plaintiffs failed to provide any proof of any financial obligation for any extraordinary medical or educational expense [44].

The issue of whether the extraordinary expenses that may be associated with caring for a child with a disability should result in the award of damages or whether the availability of public health, education and welfare resources negates such damages extends beyond the adjudication of individual cases into a debate over what the private responsibilities of parents to care for children with disabilities versus whether these costs should be assigned to defendants in a malpractice action [45].

A corollary issue is whether parents can recover expenses of caring for a child beyond the age of majority. The analysis of this issue is dependent on how individual states address the obligations of parents to support their dependent children. Some courts have allowed parents to recover post-majority expenses based on the theory that the parents’ obligation to support a child with a disability does not end when the child reaches the age of majority if they prove that the child will be dependent upon the parents beyond the age of majority. 

In *Gallagher v. Duke University* (1988), a North Carolina case, the appellate court found that the parents did have a cognizable claim for wrongful birth and could recover damages for the cost of rearing the child unmitigated by any consideration of the benefits parenthood. Additionally, the parents were allowed a claim for emotional distress. In addressing the issue of damages, the court limited recovery to extraordinary expense for the child until the death of the parents, ruling that while it is certainly understandable that the parents wish to insure that the child receives proper care after their deaths, the court cannot ignore the North Carolina law that terminates their obligation to support the child at their deaths [46].

In *Garrison v. Medical Center of Delaware* (1989), the court also disallowed the child’s action for wrongful life. However, it allowed the parents to recover special damages for the extraordinary expenses related to rearing and educating the child. In doing so, the court cited the state statute obligating the parents to provide for a dependent child [38]. The court also held that the parents stood in a fiduciary relationship and that the parents were required to account for the investment and expenditure of any amounts received as damages before the trial court [38].

However, in states where parents do not have any legal obligation to support adult children, courts have declined to allow parents to recover for post-majority extraordinary expense. For example, the Illinois Supreme Court has rejected the argument that parents of children with disabilities are entitled to damages associated with post-majority expenses [16,42]. Most recently, the court rejected the plaintiffs’ argument that public policy justifies departure from the general rule that if the common law and statutory law do not impose the obligation of support of an adult child on the parents, then a defendant in a wrongful birth action cannot be held liable for expenses that the plaintiff elects, but is not legally obligated to incur [42]. In making this ruling, the court found that holding wrongful-birth defendants liable to parents for the child’s post-majority expenses would obscure the previous distinction between wrongful birth and wrongful life. The court reiterated that, in that jurisdiction, there is no cause of action for wrongful life, because the child, while burdened, cannot be said to have suffered a legal wrong.

## 10. Emerging Issues

As noted above, many of the issues surrounding wrongful life and wrongful birth litigation are well-settled. The emergence of technologies, such as PGD, often result in some uncertainty about how such cases are conceptualized given the limitations of traditional legal constructs and the potential legal distinctions that may or may exist between the testing of *in vitro* embryos versus invasive prenatal genetic diagnosis of established pregnancies. However, ultimately, even those cases are pled and tried employing traditional legal theories, such as informed consent and negligence.

One such emerging legal issue relates to the introduction of array comparative genome hybridization (aCGH) in clinical prenatal genetic testing, which will likely result in similar questions and answers. Numerous studies have demonstrated the superiority of chromosomal microarray analysis compared with conventional karyotyping. A consequence of such testing prenatally is the identification of variations of unknown significance (VOUS). Clinicians are currently still considering issues arising from interpreting and communicating findings of uncertain or unknown clinical significance. In the United States, VOUS are routinely reported to the referring source, e.g., genetic counselor, medical geneticist, primary care physician. That professional is therefore obligated to inform the parents. The resulting decisions by the professional may give rise to allegations of malpractice depending on the decision made and whether the VOUS resulted in some form of intellectual or physical abnormality. The courts will then have to consider whether the decision by the referring healthcare professional was negligent or not, along with the myriad other issues that often arise under such causes of actions.

## 11. Cell-Free Fetal DNA-Based NIPT

In the past decade, the clinical practice of prenatal genetic testing has focused on providing services earlier in pregnancy to broader categories of women. In January, 2007, the American College of Obstetricians and Gynecologists (ACOG) revised its guidelines to recommend that fetal chromosomal screening be offered to all pregnant women, regardless of age, because of improvements in low-risk, noninvasive screening methods [47]. The guidelines also emphasize that a maternal age of 35 years should not be used as a cutoff for offering diagnostic testing. In making these recommendations, the ACOG’s Practice Bulletin “Screening for Fetal Chromosomal Abnormalities” acknowledged that in the past several years, this was already occurring widely in clinical practice [47]. The recommendation is not that all women be screened, but that all pregnant women be offered screening for aneuploidies, reflecting the ethical construct that physicians are obligated to inform patients of all of their healthcare options, but that nondirective counseling requires that the patient decides whether or not she wishes to be screened and what to do with the information if she does undergo screening [47].

In 2012, ACOG and the Society for Maternal-Fetal Medicine issued an opinion statement addressing the parameters of offering NIPT using massively parallel sequencing of maternal plasma cfDNA testing to women at increased risk for Down syndrome and trisomies 13 and 18, *i.e.*, women over age 35, those with a prior risk of pregnancy affected by a fetal trisomy, those with an increased risk of aneuploidy due to conventional serum screening or ultrasound findings or those who are carriers of a balanced Robertsonian translocation [48]. The ACOG position statement emphasizes that NIPT should be used for screening only and that positive results require confirmation with diagnostic invasive testing. The ACOG statement reflects the previous position statements issued by the National Society of Genetic Counselors and the International Society for Prenatal Diagnosis, which emphasized the importance of only offering NIPT in the context of informed consent, education and counseling by a qualified provider and that women with positive results receive genetic counseling and be afforded the option of confirmatory diagnostic testing [49].

The intent of all of these guidelines is to offer screening tests with high detection rates and low false-positive rates that also provide patients with the diagnostic options they might want to consider, with women being offered integrated or sequential screening earlier in their pregnancies. As the guidelines to offer aneuploidy screening to all women have been in place for less than a decade and the guidelines regarding NIPT have been in place for a little over two years, the long-term legal consequences have not been fully realized. Another issue is the limitations imposed by the current sensitivities of clinically available tests. While many are predicting the introduction of sequencing of noninvasively collected fetal DNA into clinical practice, NIPT is currently limited to a small number of disorders and the sex chromosomes.

One implicit purpose of any practice standard is to eliminate legal risks. The emphasis on offering all patients prenatal genetic screening may reduce or even eliminate claims of medical malpractice alleging failure to counsel about the availability of screening procedures. Similarly, cases alleging failure to counsel about the availability or to provide invasive procedures may be reduced with the provision of such procedures based on patient choice and not on specific age determinants. However, these guidelines cannot eliminate human error in the provision of screening or diagnostic tests.

The expansion of first trimester screening and, most recently, NIPT expansion of patients’ range of options about tests and procedures during their prenatal care brings discrete challenges to the process of patient education and decision-making [50]. Providing adequate prenatal counseling poses a substantial challenge given the ever-expanding range of prenatal testing options available. The frequent introduction of new testing methodologies and platforms results in the continuing need to develop the parameters of such tests for specific populations as exemplified by the current efforts to assess the performance of cfDNA testing in low-risk women [51].

Generally, professional practice guidelines emphasize that all prenatal genetic testing be offered only in the context of informed consent, education and counseling by a qualified provider. In addition, professional practice opinions encourage the provision of early and on-going counseling before and after prenatal testing to enable patients to interpret screening and test results, and some suggest that, with the increasing use of cfDNA and other noninvasive testing, preconception education be emphasized. This may be an idealized approach, as women may not present for their first prenatal visit until they are between eight and 12 weeks gestation. It is also not a realistic approach for the approximately 50% of pregnancies that are unplanned and the higher percentage of pregnant women who do not attend preconception visits with their obstetrician.

Such challenges will likely increase with the expansion of NIPT to low-risk pregnancies and the addition of cfDNA and other noninvasive forms of genetic testing into the clinical armamentarium of physicians with practices focused on managing routine pregnancies. There continues to be a shortage of genetic professionals available to meet patients pre-test and post-test educational and counseling needs, and there are concerns about the limitations of graduate and continuing training for obstetricians and gynecologists on counseling patients about Down syndrome and other aneuploidy screening procedures [50]. The screening tests require no special training for physicians who are obtaining samples from their patients. The relative ease of obtaining samples for analysis has raised questions about whether commercial vendors might provide tests to physicians or even direct-to-consumer test kits. In response, there has been a recent effort to promulgate an ethical framework that emphasizes that in the absence of government regulation, commercial testing companies and clinicians should cooperate to amend current informed consent procedures to include attention to the noninvasive nature of new testing and the potential for a broader range of results earlier in the pregnancy. These efforts also include strong recommendations that these tests are only through licensed medical providers and not directly to consumers [52].

Although NIPT is undergoing rapid and continual refinements, established screening, fetal ultrasound and invasive procedures with microarray testing allow the detection of a broad range of additional abnormalities not yet detectable by NIPT. Professional practice guidelines emphasize that patients whose NIPT results are abnormal (screen positive) or who have other factors suggestive of a chromosome or other genetic abnormality receive genetic counseling and be given the option of standard confirmatory diagnostic testing. If, however, NIPT is provided by healthcare providers without specialty training in genetics, maternal-fetal medicine or high-risk obstetrics, such care may not be as readily available or frequently considered. The potential for an uptick in medical malpractice litigation related to inadequacy of informed consent procedures (*i.e.*, the failure to advise patients about the distinctions between screening and diagnostic testing) and the failure to provide further screening and possibly invasive testing after screen positive results may be significant when the use of NIPT expands from a defined focus on high-risk pregnancies to broader-scale clinical practice [50]. As will be further discussed, ethical and legal issues resulting from NIPT will likely become more acute when testing expands to fetal whole-genome sequencing.

## 12. Restrictions on Abortion

Pregnant women and their partners undergo prenatal genetic testing for many complex and often highly individual reasons. It may be argued that prenatal genetic testing serves the general purpose of providing reassurance to pregnant women and their partners, as the majority of screening and diagnostic procedures demonstrate clinically normal genotypes. There are three other purposes of prenatal genetic testing: (1) To enable timely medical or surgical treatment of a condition before or after birth, if possible; (2) To give the parents the chance to prepare medically, emotionally, socially and in other ways for a baby with a genetic condition and resulting disabilities, or for the likelihood of fetal, neonatal or later demise; (3) To allow for the option of termination of an affected pregnancy. Federal and state laws directly and indirectly restrict abortion in the United States. Since the legalization of abortion in *Roe v. Wade* (1973), one accepted rationale cited in public opinion polls for legalized abortion is termination for fetal anomalies [53]. More recently, there has been an effort by opponents to abortion to challenge this conventional wisdom with claims that the sole purpose and outcome of prenatal genetic testing is abortion [53].

Over the past 40 years, the Supreme Court has made several changes regarding state regulation of abortions. In 1973, the Supreme Court granted women the constitutional right to terminate a pregnancy under the due process clause of the 14th Amendment [54]. *Roe v. Wade* established the following three tier framework for the regulation of abortions: (1) Tier one encompassed the first trimester and specified that states could not limit a woman’s access to abortion during this time; (2) Tier two included the time period from the end of the first trimester to the point of viability, when the fetus could survive outside of the womb, during which states can regulate abortion to protect the health of the mother; and (3) Tier three involved the time period after viability; the Court stated that during that time, states can restrict or ban abortions, so long as it is still an option for women whose lives or health are at risk [54]. Three years later, in an attempt to preserve a woman’s right to seek an abortion independently, the Supreme Court held unconstitutional portions of a Missouri statute that required a woman seeking an abortion to obtain spousal consent for the procedure [55].

In 1986, the Supreme Court further protected women’s right to obtain an abortion by ruling that a Pennsylvania statute, which required informed consent and dissemination about specific information about the abortion procedure, was an unconstitutional attempt to prevent women from making their own choices [56]. However, only six years after that decision, it was overruled by the Supreme Court in *Planned Parenthood of Southeastern Pennsylvania v. Casey* [57]. The *Casey* Court, in a plurality opinion, struck down portions of a Pennsylvania law requiring spousal notice, but upheld provisions that required pre-abortion counseling, a 24-hour waiting period and informed consent, so long as those requirements did not impose an “undue burden” on the woman [57]. More recently, in 2007, the Supreme Court upheld the Partial Birth Abortion Act of 2003, which is a federal law that prohibits what was termed in legal arguments as “partial-birth abortions” and is referred to in the medical literature as “intact dilation and extraction” [58,59]. This was a marked departure from earlier Supreme Court cases aimed at ensuring that constitutional restrictions put in place maintained a safeguard to protect the woman’s health.

The current legal landscape regarding legislative efforts to restrict access to abortion is ever-evolving. In the past few years, legislation introduced to limit abortions includes: restrictions on funding for services; banning the types or limiting the timing of procedures; prohibiting wrongful birth tort actions against physicians who claim to have withheld screening and diagnostic information that may have encouraged parents to terminate the pregnancy; imposing liability on providers through the regulation of facilities, licenses and physician conduct; and requiring patients to submit to counseling, waiting periods or measures under the guise of “informed consent” requirements, such as undergoing ultrasounds, including some measures requiring trans-vaginal procedures and viewing the resulting images. These efforts are seen by proponents of reproductive rights as an effort by legislators to abrogate women’s rights to exercise reproductive autonomy by encroaching on the practice of medicine using evidence-based standards of care.

The most recent developments in statutory abortion regulation, particularly for abortions for genetic abnormalities, have come in the form of restrictive legislation in North Dakota stating that:

“A Physician may not intentionally perform or attempt to perform an abortion with knowledge that the pregnant woman is seeking the abortion solely: (a) On account of the sex of the unborn child; or (b) Because the unborn child has been diagnosed with either a genetic abnormality or a potential for a genetic abnormality”.

[60]

Other provisions of the North Dakota Abortion Control Act also state that abortions may not be performed after a fetal heartbeat has been detected or after viability, which is statutorily set at 20 weeks gestation [61,62]. Under the aforementioned provisions, a doctor who performs an abortion after a fetal heartbeat has been detected will be charged with a class C felony, while a doctor who performs an abortion based on a “genetic abnormality” will face misdemeanor charges [60,63].

A similar bill was proposed in the Missouri House of Representatives in 2012, 2013 and 2014 [64]. The Missouri bill, Missouri Abortion Ban for Sex Selection and Genetic Abnormalities Act of 2014, states that doctors may not perform abortions if they are being sought solely “because the unborn child has been diagnosed with either a genetic abnormality or a potential for a genetic abnormality” [64]. Scholars have expressed concern that legislation such as this and the North Dakota law will force women to give birth to unwanted children, inflict suffering on those children and encourage women to withhold information from their doctors [65].

A U.S. District Court judge ruled in April, 2014, that the provision banning abortions after the detection of a fetal heartbeat was unconstitutional based on the Supreme Court’s ruling in *Roe v. Wade* [66]. Furthermore, provisions, such as those in Missouri and North Dakota, which place substantial restrictions on abortion, could face challenges in other jurisdictions particularly if they conflict with existing judicial precedents.

While prenatal genetic testing does not necessarily equate with pregnancy termination, the current legal climate for the practice of prenatal testing is further complicated by the increasingly divisive cultural and political debate over reproductive choice and access to abortion. Although legal scholars and commentators believe many of these laws will not survive court challenges, they represent a concerted effort both to stigmatize prenatal genetic testing by equating it with abortion and deprive women of the right to exercise reproductive choice and autonomy. Despite the high probability that the North Dakota law will be found unconstitutional, it is illustrative of the current political and legal climate surrounding these issues and potential significant societal attitudes toward prenatal testing. As Rachel Rebouché and Karen Rothenberg described, “Prenatal genetic testing and abortion inevitably intersect, producing discordant effects as testing becomes more common and access to abortion becomes less available” [53].

The Prenatally and Postnatally Diagnosed Conditions Awareness Act (PPDCA) provides for federal grants related to the collection of information on genetic disorders and the provision of information about raising children with Down syndrome and other prenatally or postnatally diagnosed genetic conditions [67]. It is viewed by some as demonstrating a noteworthy and admirable collaborative effort among all sides of the reproductive rights debate and disability rights activists. It is also seen by proponents as a responsible, balanced and proactive measure to address the increased use of prenatal testing resulting from the 2007 ACOG guidelines. The intent of the PPDCA is to give prospective parents accurate information so that they can make informed decisions about raising children with certain genetic disorders and is largely viewed as an attempt to encourage the dissemination of information to parents undergoing prenatal testing and support of parents caring for children with a genetic condition.

While the dictates of the PPDCA are aligned with long-standing principles of non-directive genetic counseling, in response to the PPDCA and innovations, such as NIPT, professional organizations and individual commentators have called for refinements in counseling guidelines regarding the provision of complete information to prospective parents regarding what disorders can be tested for and what results mean [68]. Given the current legal climate and the likely further expansion of efforts to restrict access to pregnancy termination services, there may be efforts by states to limit pregnant women’s access to particular genetic information until after viability or prohibit terminations based on certain diagnoses, such as for late onset disorders. In the near future, professional guidelines may be required to guide professionals in practicing non-directive counseling designed to provide information about all available options within states that impose restrictions on both patient access to information derived from prenatal genetic testing and limitations on the availability of pregnancy termination services. Table 2 provides applicable legal citations regarding how legislatures in each of the 50 United States regulate access to abortion and the actions health care providers must undertake in the provision of pregnancy termination.

**Table 2:** State statutes regarding access to abortion.

State	Abortions prohibited except in cases of life or health endangerment after	Mandatory Ultrasound	Mandatory Waiting Period	Mandatory Counseling
**Alabama**	20 Weeks (Ala. Code §§ 26-22-1 through 26-22-5)	X (Ala. Code 1975 § 26-23A-4)	48 h (2014 AL House Bill 489 (to become effective 30 days after governor signs))	Written Materials Given (Ala. Code 1975 § 26-23A-4)
**Alaska**				
**Arizona**	20 Weeks (A.R.S. § 36-2159; held unconstitutional by *Isaacson v. Horne*, 716 F.3d 1213 (9th Cir. 2013))	X (A.R.S. § 36-2156)	24 h (A.R.S. § 36-2153)	Written Materials Offered (A.R.S. § 12-719)
**Arkansas**	24 Weeks (A.C.A. § 20-16-703)		24 Hours (A.C.A. § 20-16-1103)	Written Materials Offered (A.R.S. § 20-16-903)
**California**	Viability (West’s Ann. Cal. Health & Safety Code §§ 123464 and 123468)		
**Colorado**				
**Connecticut**	Viability (C.G.S.A. § 19a-602)			
**Delaware**	20 Weeks (24 Del. C. § 1790)			
**Florida**	24 Weeks (West’s F.S.A. § 390.0111)	X (West’s F.S.A. § 390.0111)		Written Materials Offered (West’s F.S.A. § 390.0111)
**Georgia**	20 Weeks (Ga. Code Ann. § 16-12-141)		24 Hours (Ga. Code Ann. § 31-9A-3)	Written Materials Offered (Ga. Code Ann. § 31-9A-4)
**Hawaii**	Viability (HRS § 453-16)			
**Idaho**	20 Weeks (I.C. § 18-505; held unconstitutional by *McCormack v. Hiedeman*, 900 F. Supp. 2d 1128 (U.S. Dist. Ct. D. Idaho 2013))		24 Hours (I.C. § 18-609)	Written Materials Given (I.C. § 18-609)
**Illinois**	Viability (720 ILCS 510/5)			
**Indiana**	20 Weeks (IC 16-34-2-1)	X (IC 16-34-2-1.1)	18 Hours (IC 16-34-2-1.1)	Written Materials Given (IC 16-34-2-1.1)
**Iowa**	2nd Trimester (I.C.A. § 707.7)			
**Kansas**	22 Weeks (K.S.A. 65-6703)	X (K.S.A. 65-6709)	24 Hours (K.S.A. 65-6709)	Written Materials Given (K.S.A. 65-6709)
**Kentucky**	1st Trimester (KRS §§ 311.760 and 311.780)		24 Hours (KRS § 311.725)	Written Materials Offered (KSA 311.725)
**Louisiana**	20 Weeks (LSA-R.S. 40:1299.30.1)	X (LSA-R.S. 1299.35.2)	24 Hours (LSA-R.S. 1299.35.2)	Written Materials Given (LSA-R.S. 1299.35.6)
**Maine**	Viability (22 M.R.S.A. § 1598)			
**Maryland**	Viabilit (MD Code, Health-General, § 20-209)			
**Massachusetts**	24 Weeks (M.G.L.A. 112 § 12M)		24 Hours (M.G.L.A. 112 § 12S)	
**Michigan**	Viability (M.C.L.A. 750.90h)		24 Hours (M.C.L.A. 333.17015)	Written Materials Given (M.C.L.A. 333.17015)
**Minnesota**	20 Weeks (M.S.A. § 145.412; held unconstitutional by *Hodgson v. Lawson*, 542 F.2d 1350)		24 Hours (M.S.A. § 145.4242)	Written Materials Offered (M.S.A. § 145.4242)
**Mississippi**	Conception (Miss. Code Ann. § 41-41-45; to become effective in the event of *Roe v. Wade* being overruled) 20 Weeks (2014 MS House Bill 1400 (to go into effect 1 July 2014))	X (Miss. Code Ann. § 41-41-34)	24 h (Miss. Code Ann. § 41-41-33)	Written Materials Offered (Miss. Code Ann. §§ 41-41-33 and 41-41-35)
**Missouri**	Viability (V.A.M.S. 188.030)		24 h (V.A.M.S. 188.027)	Written Materials Given (V.A.M.S. 188.027)
**Montana**	Viability (MCA 50-20-109)			Written Materials Offered 24–72 hours in advance (MCA 50-20-106)
**Nebraska**	20 Weeks (Neb. Rev. St. § 28-3, 106)		24 h (Neb. Rev. St. § 28-327)	Written Materials Offered (Neb. Rev. St. § 28-327)
**Nevada**	24 Weeks (N.R.S. 442.250)			
**New Hampshire**				
**New Jersey**				
**New Mexico**	Conception (N.M.S.A. 1978, § 30-5-3; provisions held unconstitutional by *State v. Strance*, 506 P.2d 1217 (N.M. Ct. App. 1973))			
**New York**	24 Weeks (McKinney’s Penal Law § 125.05)			
**North Carolina**	20 Weeks (N.C.G.S.A. § 14-45.1)	X (N.C.G.S.A. § 90-21.85; held unconstitutional by *Stuart v. Loomis*, 2014 WL 186310 (U.S. Dist. Ct. M.D. N.C. 2014))	24 h (N.C.G.S.A. § 90-21.82)	Written Materials Offered (N.C.G.S.A. § 90-21.82)
**North Dakota**	After Heart Beat has been Detected(NDCC, 14-02.1-05.2) 20 Weeks (NDCC, 14-02.1-05.3)		24 h (NDCC, 14-02.1-02)	Written Materials Offered (NDCC, 14-02.1-02)
**Ohio**	Viability (R.C. § 2919.151; held unconstitutional by Women’s Medical Professional Corp. v. Taft, 162 F.Supp.2d 929 (U.S. Dist. Ct. S.D. Ohio 2001))		24 h (R.C. § 2317.56)	Written Materials Given (R.C. § 2317.56)
**Oklahoma**	24 Weeks (63 Okl.St.Ann. § 1-732)	X (63 Okl.St.Ann. § 1-738.3d; held unconstitutional by Nova Health Systems v. Pruitt, 292 P.3d 28 (Okla. 2012))	24 h (63 Okl.St.Ann. § 1-738.2)	Written Materials Offered (63 Okl.St.Ann. § 1-738.2)
**Oregon**				
**Pennsylvania**	24 Weeks (18 Pa.C.S.A. § 3211)		24 h (18 Pa.C.S.A. § 3205)	Written Materials Offered (18 Pa.C.S.A. § 3205)
**Rhode Island**				
**South Carolina**	2nd Trimester (Code 1976 § 44-41-20)		24 h (Code 1976 § 44-41-330)	Written Materials Offered (Code 1976 § 44-41-330)
**South Dakota**	24 Weeks (SDCL § 34-23A-5)		72 h (excludes weekends and holidays) (SDCL § 34-23A-56)	Written Materials Given (SDCL § 34-23A-10.1; portions held unconstitutional by Planned Parenthood Minn. v. Rounds, 653 F.3d 662 (8th Cir. 2011))
**Tennessee**	Viability (T.C.A. § 39-15-201)		2 days (T.C.A. § 39-15-202)	
**Texas**	20 Weeks (V.T.C.A., Health & Safety Code §§ 171.044 and 171.046)	X (V.T.C.A., Health & Safety Code § 171.012)	24 Hours (V.T.C.A., Health & Safety Code § 171.012)	Written Materials Given (V.T.C.A., Health & Safety Code § 171.012)
**Utah**	Viability (U.C.A. 1953 § 76-7-302)		72 Hours (U.C.A. 1953 § 76-7-305)	Written Materials Given (U.C.A. 1953 § 76-7-305)
**Vermont**	Bill introduced to repeal any and all prohibitions on abortion (2013 VT Senate Bill 315 (to become effective upon passing))			
**Virginia**	2nd Trimester (VA Code Ann. § 18.2-74)	X (VA Code Ann. § 18.2-76)	24 h (VA Code Ann. § 18.2-76)	Written Materials Offered (VA Code Ann. § 18.2-76)
**Washington**	Viability (West’s RCWA 9.02.110)			
**West Virginia**			24 h (W. Va. Code, § 16-2I-2)	Written Materials Offered (W. Va. Code, § 16-2I-2)
**Wisconsin**	Viability (W.S.A. 940.15)	X (W.S.A. 253.10)	24 h (W.S.A. 253.10)	Written Materials Offered (W.S.A. 253.10)
**Wyoming**	Viability (W.S. 1977 § 35-6-102)			

## 13. The Future

As Eugene Pergament notes,

“The past half-century has been witness to the application of a series of clinical and laboratory technologies that have provided remarkable insights into the human genome. The dynamism of the field of prenatal genetic diagnosis and screening is illustrated as a number of new technologies are introduced and interact: non-invasive prenatal testing (NIPT), prenatal aCGH, exome sequencing, whole genome amplification”.

[69]

As discussed above, healthcare professionals and their lawyers are already struggling with how the law responds to these technologies.

Although these innovations represent the continued progress of prenatal genetic testing, Pergament also advocates for a dramatic paradigm shift in the practice of prenatal genetic testing: “Identification of genetic and developmental disorders in the embryonic period is insufficient; there is a need to develop therapeutic interventions that correct, alleviate and/or minimize the phenotypic effects of any genetic and developmental disorder at the time of their potential genesis, namely, during embryogenesis” [69]. 

Such innovations will raise numerous ethical, legal and social questions. This includes somewhat mundane questions related to the regulation of the technologies used in the provision of these interventions. More importantly, it includes challenges to existing legal constructs regarding the responsibilities of healthcare providers and the potential rights and responsibilities of expectant parents to ameliorate the effects of an identified genetic and developmental disorder. Clinicians will have to determine their responsibilities in counseling patients regarding interventions that, while designed to minimize or even eliminate the phenotypic effect of the known genetic disorder, may not completely ameliorate the disorder or may result in yet unknown deleterious changes to the genotype. In the future, parents will likely have to confront a myriad of dilemmas far beyond what patients now must consider, to facing decisions about altering the potential development of their embryo or fetus. Undoubtedly, courts and legislatures will develop legal responses that purport to resolve some of the disputes and contradictions that surround the consequences of these technologies, while generating still more.

As Shakespeare cautioned, “and by that destiny to perform an act whereof what’s past is prologue, what to come, in yours and my discharge” [70].
